# Leucine deprivation inhibits proliferation and induces apoptosis of human breast cancer cells via fatty acid synthase

**DOI:** 10.18632/oncotarget.11626

**Published:** 2016-08-26

**Authors:** Fei Xiao, Chunxia Wang, Hongkun Yin, Junjie Yu, Shanghai Chen, Jing Fang, Feifan Guo

**Affiliations:** ^1^ Key Laboratory of Nutrition and Metabolism, Institute for Nutritional Sciences, Shanghai Institutes for Biological Sciences, The Graduate School of The Chinese Academy of Sciences, Chinese Academy of Sciences, Beijing, China

**Keywords:** leucine deprivation, breast cancer, proliferation, apoptosis

## Abstract

Substantial studies on fatty acid synthase (FASN) have focused on its role in regulating lipid metabolism and researchers have a great interest in treating cancer with dietary manipulation of amino acids. In the current study, we found that leucine deprivation caused the FASN-dependent anticancer effect. Here we showed that leucine deprivation inhibited cell proliferation and induced apoptosis of MDA-MB-231 and MCF-7 breast cancer cells. In an *in vivo* tumor xenograft model, the leucine-free diet suppressed the growth of human breast cancer tumors and triggered widespread apoptosis of the cancer cells. Further study indicated that leucine deprivation decreased expression of lipogenic gene FASN *in vitro* and *in vivo*. Over-expression of FASN or supplementation of palmitic acid (the product of FASN action) blocked the effects of leucine deprivation on cell proliferation and apoptosis *in vitro* and *in vivo*. Moreover, leucine deprivation suppressed the FASN expression via regulating general control non-derepressible (GCN)2 and sterol regulatory element-binding protein 1C (SREBP1C). Taken together, our study represents proof of principle that anticancer effects can be obtained with strategies to deprive tumors of leucine via suppressing FASN expression, which provides important insights in prevention of breast cancer via metabolic intervention.

## INTRODUCTION

Breast cancer is the leading type of cancer in women, accounting for 25% of all cases [1]. Usually, breast cancer is treated with surgery, medicine and radiation [2]. For a long time, researchers have mounting interest in treating cancer with dietary manipulation of branched-chain amino acids (BCAAs) [3–6]. Leucine, isoleucine and valine are the three BCAAs. BCAAs participate in a wide variety of metabolic pathways. It is now recognized that they are also critical regulators of many cell signaling pathways [7].

Researchers in amino acids therapy usually favor the idea of selective BCAA supplementation in the hope of overall benefit to the host with hopefully little or no advantage conferred to the tumor [4]. This is because cancer patients often experience cachexia. A number of studies have noted widespread decreases in circulating total amino acids in cachectic patients [8, 9]. In this state, BCAAs supplementation has been used to stimulate protein synthesis. However, some studies suggest caution against the clinical use of BCAAs supplementation. For example, Liu et al. showed that leucine supplementation enhanced pancreatic cancer growth in lean and overweight mice [10]. In addition, some research reported that serum leucine concentration was significantly higher [11] or remain the same [12, 13] in breast cancer patients compared with controls. These studies suggest a complex role of BCAAs in cancer and needs further investigation.

By contrast, some studies focus on selective BCAA depletion, with a view to injuring the tumor to a greater extent than the host. A previous study demonstrated that leucine deprivation caused the caspase-dependent apoptosis of melanoma cells *in vitro* but dietary leucine deprivation on its own did not significantly affect tumor size *in vivo* [14]. The goal of our current study is to investigate the effect of leucine deprivation on breast cancer cells *in vitro* and *in vivo* and to elucidate the underlying mechanisms.

## RESULTS

### Leucine deprivation reduces viability of cancer cells

**Figure 1:**
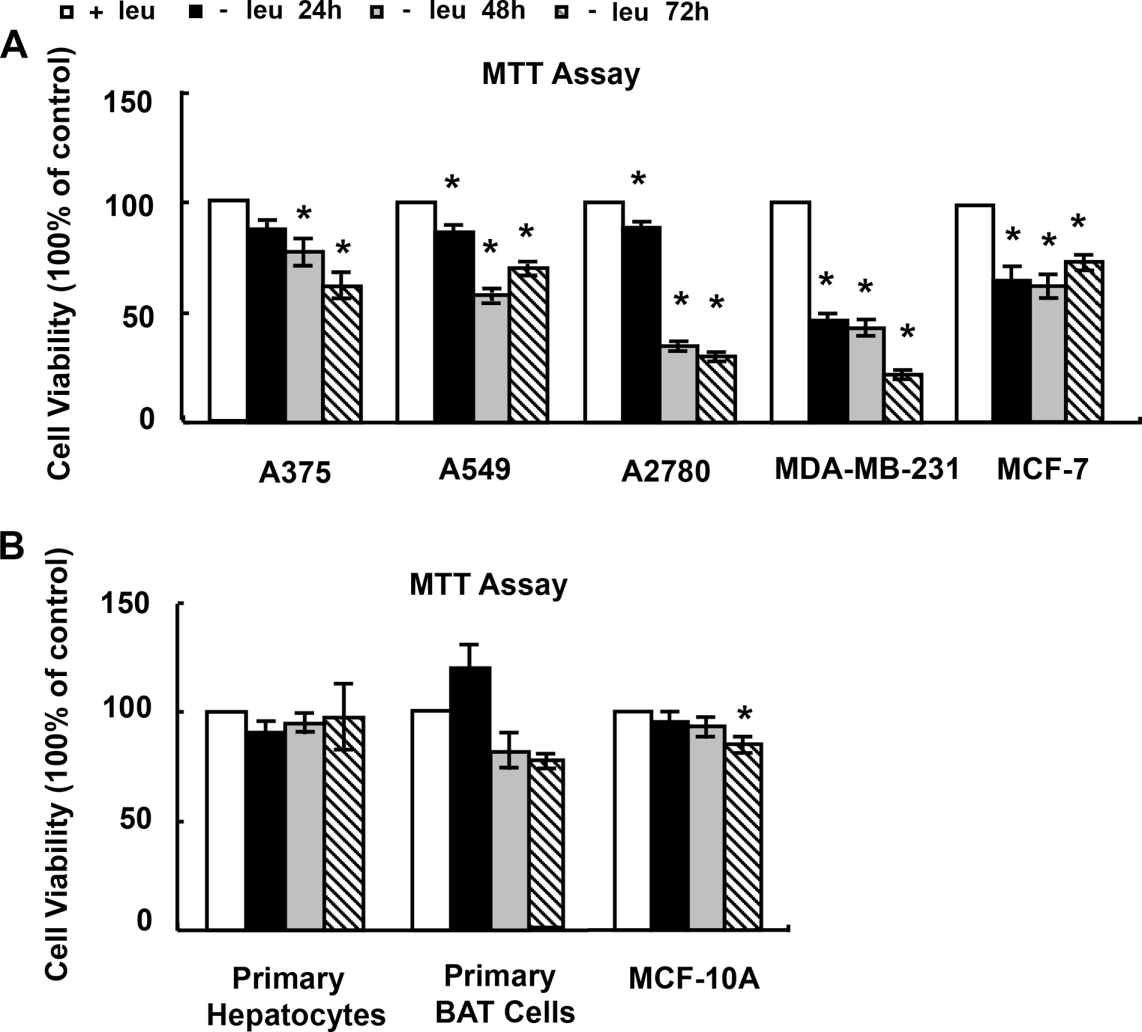
The effect of leucine deprivation on cell viability (**A** and **B**) Cells were incubated in control (+leu) or leucine-deficient (−leu) medium for indicated time, followed by MTT assay. The data are means ± SEMs (*n* = 5–6). Statistical analysis was performed using the two-tailed Student *t* test for the effects of the (−) leu medium vs. control medium (*p < 0.05).

We first determined the effect of leucine deprivation on viability of cancer cells. Four types of cancer cells were tested: malignant melanoma A375, lung caner A549, ovarian carcinoma A2780 and breast cancer MCF-7 and MDA-MB-231 cells. The treatment of these cells with (−) leu medium resulted in a significant reduction in cell viability as assessed by the MTT assay (Figure 1A). Among these cells, MDA-MB-231 cells were most sensitive to leucine deprivation (Figure 1A). We further investigated the cytotoxicity of leucine deprivation on normal cells, including primary hepatocytes, brown adipose tissue (BAT) and MCF-10A cells. We didn't find significant reduction in cell viability on primary hepatocytes and BAT cells treated with (−) leu medium (Figure 1B). MCF-10A cell viability was decreased about 15% by leucine deprivation for 72 h (Figure 1B).

### Leucine deprivation inhibits breast cancer cell proliferation

**Figure 2:**
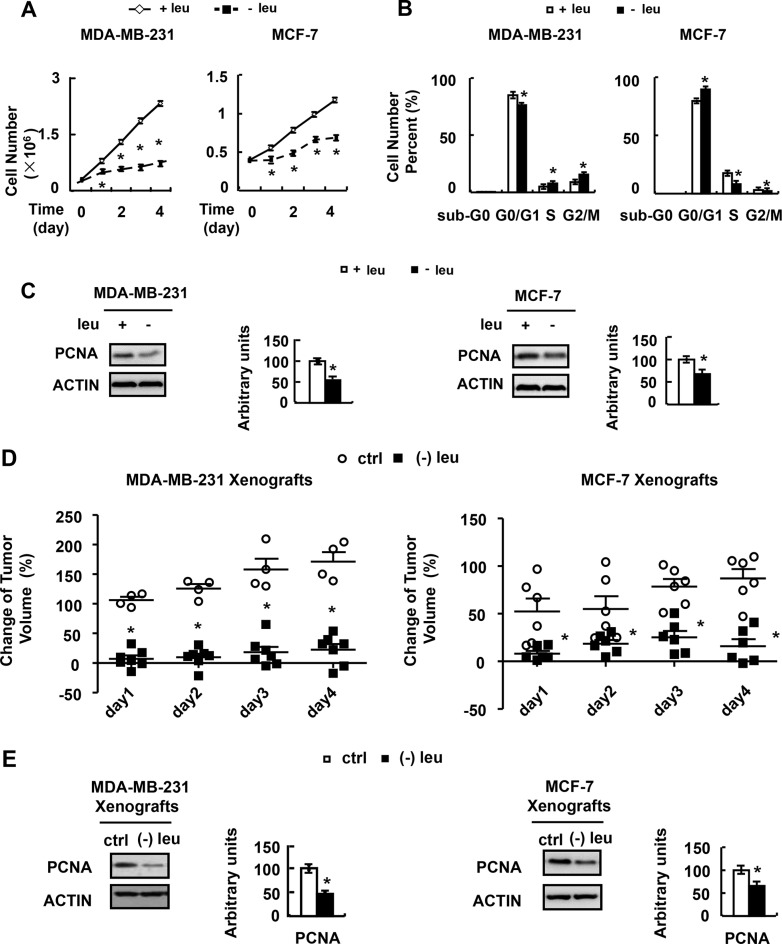
Leucine deprivation inhibits breast cancer cell proliferation (**A**) MDA-MB-231 or MCF-7 cells were seeded in 12-well plates and incubated in control (+leu) or leucine-deficient (−leu) medium. The cells were harvested at indicated time and cell numbers were counted under a microscope. (**B** and **C**) MDA-MB- 231 or MCF-7 cells were incubated in control (+leu) or leucine-deficient (−leu) medium for 48h, followed by cell cycle distribution assay in B, examination of PCNA abundance in C. (**D** and **E**) Nude mice bearing tumors were fed with control (ctrl) or (−) leu diet, followed by measurement of the tumor volume in D, examination of PCNA abundance in E. Means ± SEMs shown are representative of at least three independent experiments *in vitro* and two independent *in vivo*. Statistical analysis was done using the two-tailed Student *t* test for the effects of (−) leu vs. the control treatment (*p < 0.05).

We next analyzed the growth rate of MDA-MB-231 and MCF-7 cells incubated with (−) leu medium. The growth rate of these cells was significantly reduced when grown in (−) leu medium (Figure 2A). Consistent with this observation, the expression of PCNA, a proliferation marker, was decreased by leucine deprivation in MDA-MB-231 and MCF-7 cells (Figure 2C).

As there was a significant growth-inhibitory effect of leucine deprivation, we explored whether leucine deprivation had any inhibitory effect on cell cycle progression. Depletion of leucine resulted in a reduction of G1 population, and an accumulation of cells in the S-phase of cell cycle in MDA-MB-231 cells (Figure 2B). However, leucine deprivation resulted in a decrease of S population of MCF-7 cells (Figure 2B).

To assess the effect of leucine deprivation on the tumorigenicity *in vivo*, MDA-MB-231 and MCF-7 cells were injected into mammary glands of nude mice to establish tumor xenografts. Then these mice were fed with control or leucine-deficient diet. We found that the xenografts grew rapidly in mice fed with control diet (Figure 2D). In contrast, leucine deprivation significantly inhibited *in vivo* tumor growth of MDA-MB-231 and MCF-7 cells (Figure 2D). In consistent with the observation *in vitro*, the PCNA protein abundance was decreased in tumors by leucine deprivation (Figure 2E).

### Leucine deprivation induces apoptosis of human breast cancer cells

**Figure 3:**
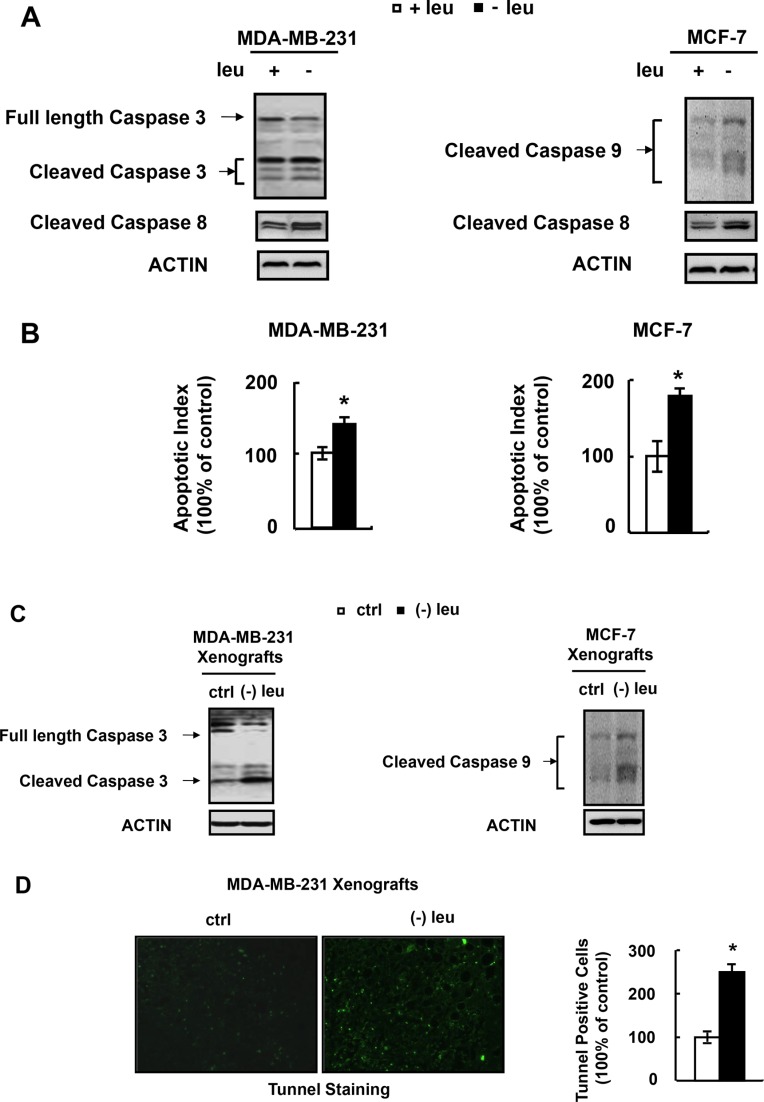
Leucine deprivation induces apoptosis of human breast cancer cell (**A** and **B**) MDA-MB-231 or MCF-7 cells were incubated in control (+leu) or leucine-deficient (−leu) medium for 48h, followed by determination of cleaved caspases in A and annexin-V assay in B. (**C** and **D**) Nude mice bearing tumors were fed with control (ctrl) or (−) leu diet, for 4 days, followed by examination of cleaved caspases in C and *in situ* tunnel assay in D. Means ± SEMs shown are representative of at least three independent experiments *in vitro* or at least two independent *in vivo*. Statistical significance was calculated using the two-tailed Student *t* test for the effects of (−) leu vs. the control treatment (*p < 0.05).

A previous study demonstrated that leucine deprivation induced the caspase-dependent apoptosis of melanoma cells [14]. We investigated whether leucine deprivation had similar effect on breast cancer cells. Firstly, we determined the expression of apoptosis-related protein cleaved-caspase-3 and cleaved-caspase-8 in MDA-MB-231 cells grown in (−) leu medium. We found that the levels of cleaved-caspase-3 and cleaved-caspase-8 in MDA-MB-231 cells were elevated under leucine deprivation (Figure 3A). Similarly, the levels of cleaved-caspase-8 and cleaved-caspase-9 in MCF-7 cells were increased by leucine deprivation (Figure 3A). As MCF-7 cells are negative for caspase-3 expression, we detected cleaved-caspase 9 instead. We also determined whether leucine deprivation could trigger apoptosis of these cells by Annexin-V assay and found that leucine-deficiency led to apoptosis of both MDA-MB-231 and MCF-7 cells (Figure 3B). Consistent with the *in vitro* data, leucine-deprived diet increased the cleaved caspase proteins of xenografts of MDA-MB-231 and MCF-7 cells (Figure 3C). We stained the MDA-MB-231 xenografts with a TUNEL kit. The results showed that the tumors from mice fed with leucine-deficient diet had more TUNEL-positive staining (Figure 3D).

### Fatty acid synthase (FASN) mediates the effect of leucine deprivation on breast cancer cells

**Figure 4:**
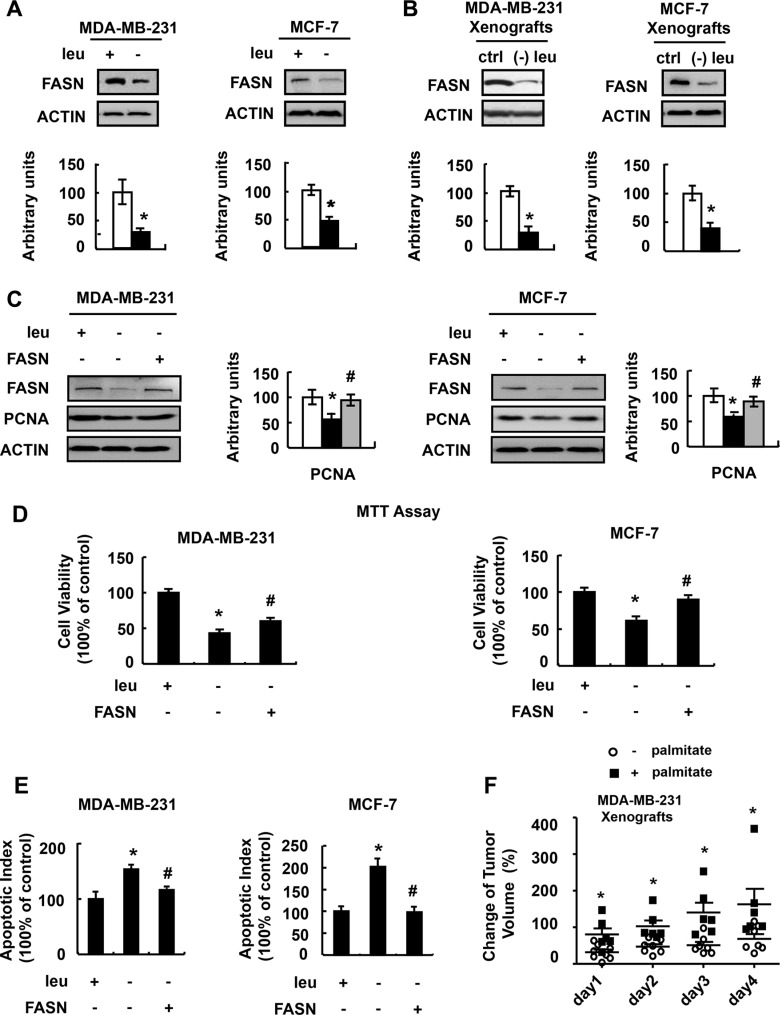
FASN mediates the effect of leucine deprivation on breast cancer cells (**A**) MDA-MB-231 or MCF-7 cells were incubated in control (+leu) or leucine- deficient (−leu) medium for 48 h, followed by determination of FASN protein abundance. (**B**) Nude mice bearing tumors were fed a control (ctrl) or (−) leu diet for 4 days, followed by examination of FASN protein abundance. (**C**–**E**) FASN- over-expressing stable cells (+FASN) or control cells (−FASN) were incubated in control (+leu) or leucine-deficient (-leu) medium for 48 h, followed by determination of FASN expression in C, examination of cell viability in D and Annexin-V assay in E. (**F**) Nude mice bearing MDA-MB-231 tumor xenografts were orally supplemented with palmitic acid (+palmitate) or control solution (−palmitate) when fed with (−) leu diet, followed by measurement of the tumor volume at the indicated time point. Means ± SEMs shown are representative of at least three independent experiments *in vitro* or at least two independent *in vivo*. Statistical significance was calculated using the two-tailed Student *t* test for the effects of (−) leu vs. the control treatment (*p < 0.05) in A and B, with vs. without palmitic acid supplementation (*p < 0.05) in F, or using the one-way ANOVA followed by the Student- Newman-Keuls (SNK) test for the effects of (−) leu vs. the control treatment (*p < 0.05) in C–E, with or without FANS over-expression in (−) leu group (^#^*p* < 0.05) in C–E.

We next explored the possible mechanism underlain. We focused on protein FASN. FASN is a key lipogenic enzyme catalyzing the terminal steps in the de novo biogenesis of fatty acids and plays important role in cancer biology [15–18]. The main function of FASN is to catalyze the synthesis of palmitate C16:0 from acetyl-CoA and malonyl-CoA. We found that leucine deprivation decreased FASN expression and activity in the liver and adipose tissue of mice [19]. So we proposed that FASN might mediate the effect of leucine deprivation on breast cancer cells. As expected, FASN protein abundance was significantly decreased in MDA-MB-231 and MCF-7 cells grown in (−) leu medium (Figure 4A). Similar results were obtained in tumor of nude mice fed with leucine-deficient diet (Figure 4B). In addition, the concentration of C16:0 was lower in the MDA-MB-231 xenografts of nude mice fed with leucine- deficient diet, compared with those from mice maintained on a control diet (Supplementary Figure S1).

If decreased FASN regulates cell viability under leucine deprivation, upregulation of FASN should block the effect of leucine deprivation on reducing cell viability. As predicted, over-expression of FASN (Figure 4C) increased viability of MDA-MB-231 and MCF-7 cells (Figure 4D). In addition, the leucine deprivation-induced apoptosis was reduced in these cells (Figure 4E). To demonstrate the importance of FASN in decreased tumor growth under leucine deprivation *in vivo*, leucine-deprived mice were orally provided with palmitic acid, the product of FASN action, or control vehicle. Palmitic acid supplementation resumed the xenograft growth (Figure 4F).

### Leucine deprivation decreases FASN expression via general control non-derepressible (GCN)2 and sterol regulatory element-binding protein 1C (SREBP1C) signaling

**Figure 5:**
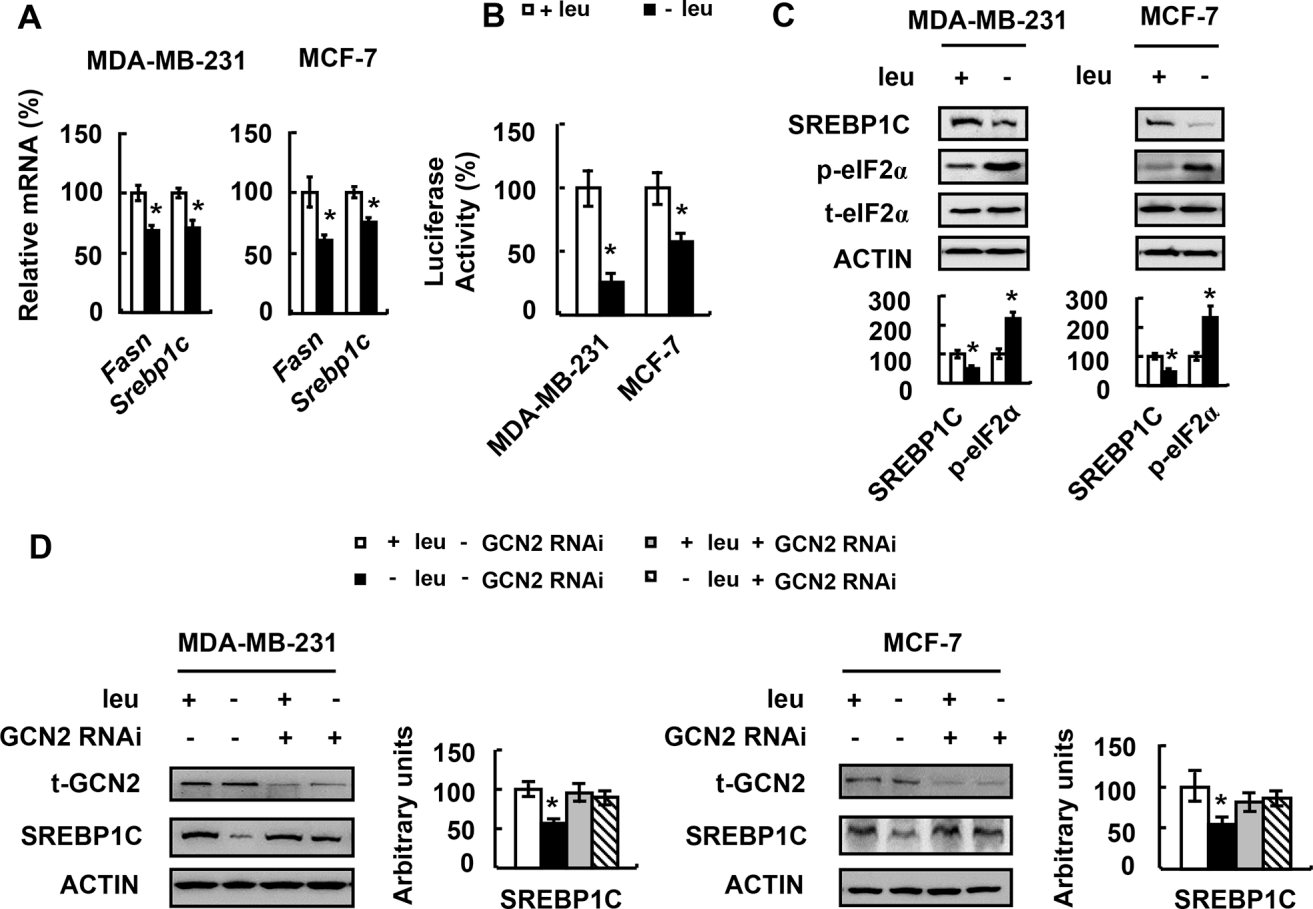
Leucine deprivation decreases FASN expression via GCN2/SREBP1C signaling (**A** and **C**) MDA-MB-231 or MCF-7 cells were incubated in control (+leu) or leucine-deficient (−leu) medium for 48 h, followed by examination of the corresponding mRNA in A and proteins in C. (**B**) Cells expressing the *Fasn* promoter vector were incubated in control (+leu) or leucine-deficient (−leu) medium for 48 h, followed by examination of luciferase activity. (**D**) Cells with (+GCN2 RNAi) or without (−GCN2 RNAi) GCN2 knockdown were incubated in control (+leu) or leucine-deficient (−leu) medium for 48 h, followed by examination of the corresponding proteins. Statistical significance was calculated using the two-tailed Student *t* test for the effects of (−) leu vs. the control treatment (*p < 0.05) in A–C, or using the one-way ANOVA followed by the Student- Newman-Keuls (SNK) test for the effects of (−) leu vs. the control treatment (*p < 0.05) in D.

To explore the underlying mechanism of FASN protein abundance reduction, we first examined *Fasn* mRNA abundance in MDA-MB-231 and MCF-7 cells cultured in (−) leu medium. *Fasn* mRNA was decreased by leucine deprivation in both cell lines (Figure 5A). We further determined the effect of leucine deprivation on *Fas*n promoter in MDA-MB-231 and MCF-7 cells. We found that leucine deprivation decreased *Fasn* promoter activity (Figure 5B).

SREBP1C is a transcription factor that directly binds to *Fasn* promoter and regulates *Fasn* mRNA abundance [20]. We found that SREBP1C mRNA and protein abundance was decreased by leucine deprivation in MDA-MB-231 and MCF-7 cells (Figure 5A and 5C). This suggests that leucine deprivation reduces *Fasn* promoter activity by decreasing SREBP1C expression.

GCN2 is a serine protein kinase that functions as a sensor for amino acid deprivation [21, 22]. To know whether GCN2 was invovled in the regulation of SREBP1C expression under leucine deprivation, we determined the phosphorylation of eIF2α, a target of GCN2 [23], in cells grown in (−) leu medium. Consistent with previous studies, eIF2α phosphorylation was increased (Figure 5C). Furthermore, decreased SREBP1C protein abundance by leucine deprivation was not observed in GCN2-knocking down MDA-MB-231 and MCF-7 cells (Figure 5D).

## DISCUSSION

There is a great interest in the roles of BCAAs in tumorigenesis and their potential use in cancer therapies. In this study, we observed that 1) leucine deprivation reduces cell viability, inhibits cell proliferation and induces apoptosis of breast cancer cells *in vitro*; and 2) dietary leucine deprivation significantly inhibited *in vivo* tumor growth of breast cancer cells. In the current study, we observed that mice maintained on a leucine deficient diet reduced their food intake by 20% (Supplementary Figure S2). By using pair-fed mice as a control, we demonstrate that the reduced tumor growth was caused primarily by deficiency of leucine, rather than the reduction in food intake.

We also tested the effect of other three essential amino acids (valine, isoleucine and phenylalanine) and one non essential amino acids (glycine) deprivation on MDA-MB-231 cell viability (Supplementary Figure S3). Among the four amino acids tested, only valine deprivation had similar effect as leucine-deficiency. These results reveal that individual amino acids have different effects on cancer cell viability.

In addition, we tested the effect of leucine deprivation on cell viability of primary hepatocytes, BAT and MCF-10A cells. Leucine deprivation for 48 h didn't decrease viability of these cells, suggesting potential application of this diet in treating breast cancer. Furthermore, we did dose-dependent study and found that the viability of MDA-MB-231 cells was reduced when the leucine amount was 10% of normal concentration (Supplementary Figure S4). Clinic use of this diet, however, is still premature, because the safety of short- and long-term leucine deprivation in humans has not been examined. Determining the optimal concentration of leucine and the duration of therapeutic leucine-deprived diets will be important in future studies.

It has been reported that amino acids deprivation inhibits cell cycle progression [24, 25]. However, amino acids deficiency mediates distinct checkpoints in different cells [26, 27]. There have been few reports on the effect of leucine deprivation on cell cycle regulation. We found that leucine deprivation resulted in a reduction of G1 population, as well as an accumulation of cells in the S-phase of cell cycle in MDA-MB-231 cells. However, leucine deprivation resulted in a reduction of S population in MCF-7 cells. The reason for this distinctive phase arrest by leucine deprivation may be that MDA-MB-231 cells are triple negative while MCF-7 cells are estrogen and progesterone receptor positive. These results demonstrate that nutrient sensing metabolic checkpoints are regulated differently in diverse cancer cells.

Upregulation of FASN represents a nearly-universal phenotypic alteration in most human malignancies including breast cancer [28]. Studies show that FASN inhibition induces cell-cycle arrest, leading to decrease in tumor cell proliferation and apoptosis [29]. Here we observed decreased FASN protein abundance under leucine deficiency in breast cancer cells. Thus, the effect of leucine deprivation on cell proliferation and apoptosis is likely mediated by its inhibiting effect on FASN expression. The importance of leucine deficiency- dependent repression of FASN was further demonstrated *in vitro* and *in vivo* by FASN over-expression or supplementation with palmitic acid, the product of FASN action, respectively. In the current study, we tested the effect of leucine deprivation on four types of cancer cells: malignant melanoma A375, lung caner A549, ovarian carcinoma A2780 and breast cancer MCF-7 and MDA-MB-231 cells. Among these cells, MDA-MB-231 cells are most sensitive to leucine deprivation. However, this is not because the FASN protein abundance is higher in MDA-MB-231 than that in other cells examined (Supplementary Figure S5). The molecular mechanism remains to be explored.

To explore the underlying mechanism for FASN protein abundance reduction, we detected the effect of leucine deprivation on *Fasn* mRNA abundance and promoter activity. Our results indicate that lecuine deprivation decreases FASN expression at transcriptional level. Then we focused on transcription factors that can bind to *Fasn* promoter and regulates *Fasn* transcription. One of these factors is SREBP1C. We found that SREBP1C expression was decreased upon leucine deprivation. This suggests that leucine deprivation inhibits *Fasn* transcription through suppressing SREBP1C expression.

To look for upstream regulators for SREBP1C, our attention was drawn to the amino acid sensor GCN2, a kinase that is activated by uncharged tRNA in response to deprivation of essential amino acids [21, 22]. We previously demonstrated that GCN2 regulated hepatic lipid metabolism during leucine deprivation [30], suggesting a possible involvement of GCN2 in regulating SREBP1C expression in breast cancer cells under leucine deprivation. Consistent with this hypothesis, decreased SREBP1C protein abundance by leucine deprivation was not observed in GCN2-knockdown cells. This suggests that GCN2 functions as an upstream regulator of SREB1C under leucine deprivation. The mechanism by which GCN2 regulates SREBP1C expression requires further investigation.

Sheen et al. [14] found that leucine deprivation caused caspase-dependent apoptosis of melanoma cells *in vitro* but dietary leucine deprivation on its own did not significantly affect tumor size *in vivo*. Consistent with this study, we also observed that leucine deficiency induced apoptosis of breast cancer cells. Evidence in Sheen's study suggests that leucine deprivation triggers apoptosis because, unlike in other cell types, it does not inhibit the mTORC1 pathway and, thus, does not activate autophagy. The hyperactivation of the RAS-MAPK pathway that is a common occurrence in melanoma cells contributes to the insensitivity of mTORC1 to leucine deprivation. Singh et al. found that leucine restriction did not decrease mTOR signaling in any of the 8 breast cancer cell lines tested including MDA-MB-231 and MCF-7 [31], suggesting that the leucine deficiency-induced apoptosis of breast cancer cells may have similar mechanisms. However, in contrast to Sheen's study, we observed that dietary leucine deprivation significantly inhibited *in vivo* tumor growth of MDA-MB-231 and MCF-7 cells. The distinct effect of leucine deprivation among different cancer cells could be due to the unique characteristic of each cancer.

## MATERIALS AND METHODS

### Plasmids, cell culture and treatments

Malignant melanoma A375, lung caner A549, ovarian carcinoma A2780 and breast cancer MCF-7 cells were originally obtained form the American Type Culture Collection (ATCC, Manassas, VA). Breast cancer cells MDA-MB-231 and MCF-10A were purchased from the Cell Bank Type Culture Collection of Chinese Academy of Sciences, Shanghai, China. All cells were cultured according to the ATCC instructions. Primary hepatocytes [32] and BAT cells [33] were isolated as previously described. Control (complete amino acid), leucine-deficient, valine-deficient, isoleucine-deficient, phenylalanine-deficient or glycine-deficient medium were prepared by adding all the components of regular DMEM or lacking the indicated amino acid. It's well-known that transcription from the asparagine synthetase (ASNS) gene is increased in response to leucine deprivation [34]. As expected, *Asns* mRNA was increased in cells grown in (−) leu medium (Supplementary Figure S6). All the media were without FBS or NBS. The cDNAs of human fatty acid synthase (FASN) was provided by Dr. Massimo Loda (Dana-Farber Cancer Institute, Boston, MA). The FASN over-expressing stable cell lines were obtained by transfecting pbabe-puro-FASN vector into MDA-MB-231 or MCF-7 cells and selecting for stable clones in the presence of 0.5 μg/ml puromycin. The *Fasn* promoter was provided by Dr. Tim Osborne (Sanford-Burnham Medical Research Institute, Orlando, FL). The double-stranded siRNA targeting GCN2 was from GenePharma (Shanghai, China). The sequence is 5′-CTGGATGGATTAGCTTATAT-3′. Cells were transfected with siRNA using X-tremeGene siRNA Transfection Reagent (Roche Diagnostics, Mannheim, Germany).

### Detection of cell viability, proliferation, cell cycle and apoptosis

MTT and analysis of apoptosis in cell were conducted as previously described [35]. The cell cycle distribution was determined using a FACSAria instrument. Tunel assay of tumor sections was detected using the *In Situ* Cell Death Detection Kit (Roche, Rotkreuz, Switzerland) according to the manufacturer's instructions.

### Western blot analysis

Western blot analysis was performed as previously described [36]. Primary antibodies against PCNA and SREBP1C were from Santa Cruz Biotechnology, Santa Cruz, CA; antibodies against FASN were from BD Biosciences, Franklin Lakes, NJ; antibodies against cleaved-caspase 3, cleaved-caspase 8, cleaved-caspase 9, p-eIF2α, t-eIF2α and GCN2 were from Cell Signaling Technology, Beverly, MA.

### RNA isolation and relative quantitative RT-PCR

RNA isolation and relative quantification RT-PCR were performed as described previously [37]. The sequences of primers used for RT-PCR are listed in Supplementary Figure S7.

### Luciferase assay

MDA-MB-231 and MCF-7 cells were co-transfected with the internal control vector pRL-*Renilla* and *Fasn* promoter using Effectene Transfection Reagent (Qiagen, Hilden, Germany), followed by incubation with control or (−) leu medium for 48 h. The firefly and renilla luciferase activities were assayed using Dual-Glo Luciferase assay system (Promega, Madison, WI).

### Xenograft tumor assay and treatment protocols

Female BALB/c nude mice (6-week old) were obtained from Shanghai Laboratory Animal Co., Ltd. (SLAC, Shanghai, China) and maintained in pathogen-free conditions. MDA-MB-231 or MCF-7 cells were harvested and resuspended in PBS containing 50% (v/v) Matrigel (BD Biosciences, Bedford, MA). The cell suspension (2 × 10^6^ MDA-MB-231 cells or 10^7^ MCF-7 cells in 0.1 ml PBS per mouse) was injected into the mammary glands of female nude mice. At the start of the feeding experiments, mice bearing tumors were acclimated to a control diet for 7 days and then randomly divided into control- and (−) leu diet groups, with free access to control and (−) leu diet, respectively, for 4 days. In addition, a pair-fed group was included to distinguish possible influences from a reduction in food intake previously observed in the (−) leu group. The pair-fed mice were provided with 20% less food compared with mice in the control diet group. This percentage was determined by our observation in the current study that, on average, mice maintained on a leucine-deficient diet consumed 20% less food compared with mice maintained on a control diet. Food intake and body weight were recorded daily. The tumor growth of the mice was monitored every day. Tumor volume (V) was calculated using the values of the largest (A) and the smallest (B) diameter according to the formula V = 0.5 × AB^2^ [38]. Change of tumor volume = (volume of the indicated day –day 0 volume)/day 0 volume. Control (nutritionally complete amino acid) and (−) leu (leucine-deficient) diets were obtained from Research Diets, Inc. (New Brunswick, NJ). All diets were isocaloric and compositionally the same in terms of carbohydrate and lipid component. For oral administration, 120 mg palmitic acid (Sigma) was dissolved in 1 ml control solution containing 80 % ethanol, 10 % Tween 80, and 10% polyethylene glycol. Mice bearing palpable tumors were fed with (−) leu diets when orally supplemented with palmitic acid (300 mg/kg body weight) or control solution every afternoon for 4 days. These experiments were conducted in accordance with the guidelines of the Institutional Animal Care and Use Committee of the Institute for Nutritional Sciences, Shanghai Institute for Biological Sciences, Chinese Academy of Sciences.

### Measurement of fatty acid composition

The tumor (weighing 10 mg) was first homogenized in ice cold PBS added with internal standard. Fatty acid composition was measured as previously described [39].

### Statistics

All data are expressed as mean ± SEM. Significant differences were assessed either by two-tailed Student *t*-test or one-way ANOVA followed by the Student-Newman-Keuls (SNK) test. *P* < 0.05 was considered statistically significant.

## SUPPLEMENTARY MATERIALS FIGURES


