# Supplementary material for: Leucine deprivation inhibits proliferation and induces apoptosis of human breast cancer cells via fatty acid synthase
Source: Oncotarget. 2016 Aug 26;7(39):63679–89. doi: 10.18632/oncotarget.11626 (PMC5325395; doi:10.18632/oncotarget.11626)
Supplement: Supplementary file 1 [file oncotarget-07-63679-s001.pdf]

Leucine deprivation inhibits proliferation and induces apoptosis of human breast cancer cells via fatty acid synthase

Supplementary Materials

Tumor fatty acid composition in leucine-deprived mice

fatty acid	control diet	(-) leu diet	p	fatty acid	control diet	(-) leu diet	p
14:0	0.70±0.03	0.65±0.08	0.603	20:1n9	0.60±0.03	0.57±0.04	0.636
15:0	0.18±0.01	0.13±0.01*	0.026	20:2n6	0.98±0.31	0.63±0.05	0.308
16:0	18.9±0.49	16.9±0.64*	0.047	20:3n6	1.75±0.11	1.2±0.15*	0.02
16:1n7	1.3±0.17	2.88±0.57*	0.029	20:4n6	14.96±0.95	14.39±1.16	0.718
16:1n9	1.17±0.13	0.98±0.10	0.272	20:5n3	0.24±0.03	0.12±0.01*	0.009
17:0	0.35±0.02	0.30±0.02	0.062	22:0	0.54±0.05	0.36±0.03*	0.011
18:0	17.13±0.63	15.03±1.00	0.126	22:4n6	4.25±0.38	3.08±0.17*	0.024
18:1n7	3.06±0.15	2.61±0.09*	0.026	22:5n3	0.53±0.03	0.44±0.05	0.173
18:1n9	16.85±0.70	19.99±1.62	0.132	22:5n6	1.53±0.14	1.04±0.14*	0.038
18:2n6	9.04±0.78	11.29±0.52*	0.036	22:6n3	3.57±0.25	3.17±0.32	0.363
18:3n3	0.08±0.08	0.08±0.01	0.968	23:0	0.51±0.05	0.57±0.04	0.377
18:3n6	0.18±0.02	0.15±0.01	0.146	24:0	1.08±0.09	0.82±0.06	0.051
20:0	0.40±0.03	0.35±0.02	0.26	24:1n9	0.62±0.03	0.47±0.05*	0.045

Data is % of total fatty acids.

Supplementary Figure S1: Tumor fatty acid composition in leucine-deprived mice. Nude mice bearing MDA-MB-231 xenografts were fed with control (ctrl) or (−) leu diet for 4 days. Means ± SEMs shown are representative of at least two independent experiments *in vivo*. Statistical significance was calculated using the two-tailed student t test for the effects of (−) leu vs. the control treatment (*$p < 0.05$).

A **Body weight change and food intake in leucine-deprived mice**

	Mice with MDA-MB-231 Xenografts		Mice with MCF-7 Xenografts	
	ctrl	(-) leu	ctrl	(-) leu
body weight change (%)	0.52±2.59	-13.38±0.85*	0.9±1.08	-13.29±0.65 *
food intake (g/day)	4.52±1.53	3.58±0.23 *	4.37±0.67	3.42±0.06 *

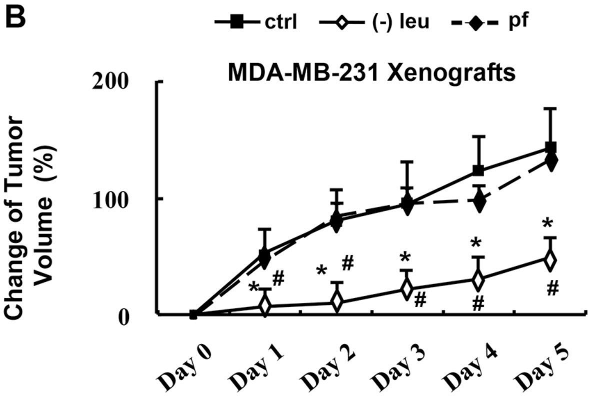

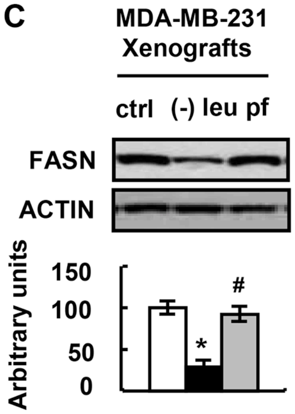

Supplementary Figure S2: The effect of leucine-deficient diet on body weight and food intake. (**A**) Nude mice bearing MDA-MB-231 or MCF-7 xenografts were fed with control (ctrl) or (−) leu diet for 4 days. (**B** and **C**) Nude mice bearing MDA-MB-231xenografts were fed with control (ctrl), (−) leu or pair-fed (pf) diet for 5 days, followed by measurement of the tumor volume at the indicated time point in B, examination of FASN protein abundance in C. Means ± SEMs shown are representative of at least two independent *in vivo* experiments. Statistical significance was calculated using the two-tailed student *t* test for the effects of (−) leu vs. control diet ($*p < 0.05$) in A, or using the one-way ANOVA followed by the Student- Newman-Keuls (SNK) test for the effects of (−) leu vs. the control treatment ($*p < 0.05$) or (−) leu diet vs. pair-fed diet ($^{\#}p < 0.05$) in B and C.

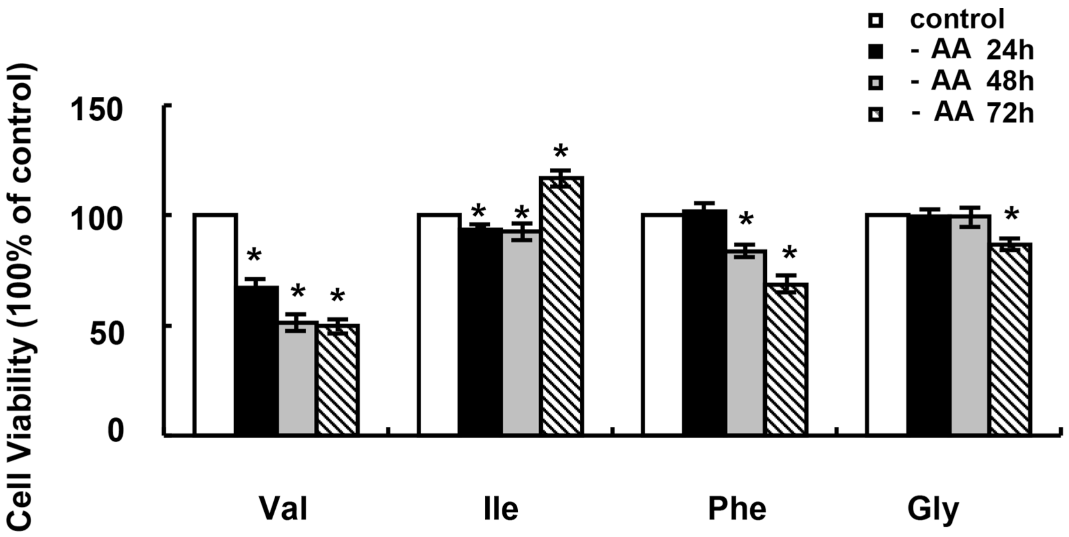

Supplementary Figure S3: The effect of amino acid deprivation on cell viability. MDA-MB-231 cells were incubated in control, valine-deficient (−Val), isoleucine-deficient (−Ile), phenylalanine-deficient (−Phe) or glycine-deficient (−Gly) medium for indicated time, followed by MTT assay. Statistical significance was calculated using the two-tailed Student *t* test for the effects of the amino acid-deficient medium vs. control medium (*$p < 0.05$).

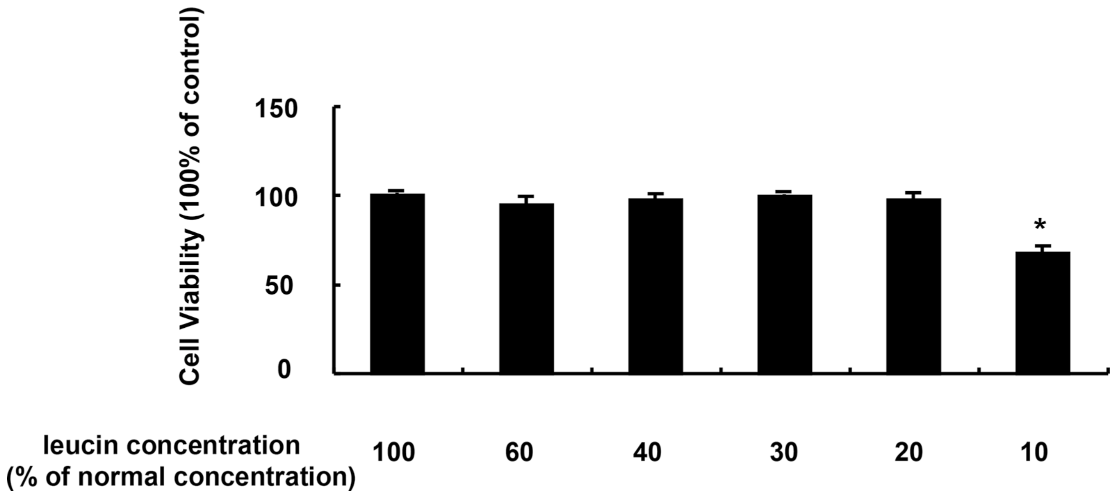

Supplementary Figure S4: The effect of different leucine concentration medium on cell viability. MDA-MB-231 cells were incubated in medium with different leucine concentration for 48 h, followed by MTT assay. Means ± SEMs shown are representative of at least three independent experiments *in vitro*. Statistical significance was calculated using the two-tailed Student *t* test for the effects of the different leucine concentration medium vs. control medium (*$p < 0.05$).

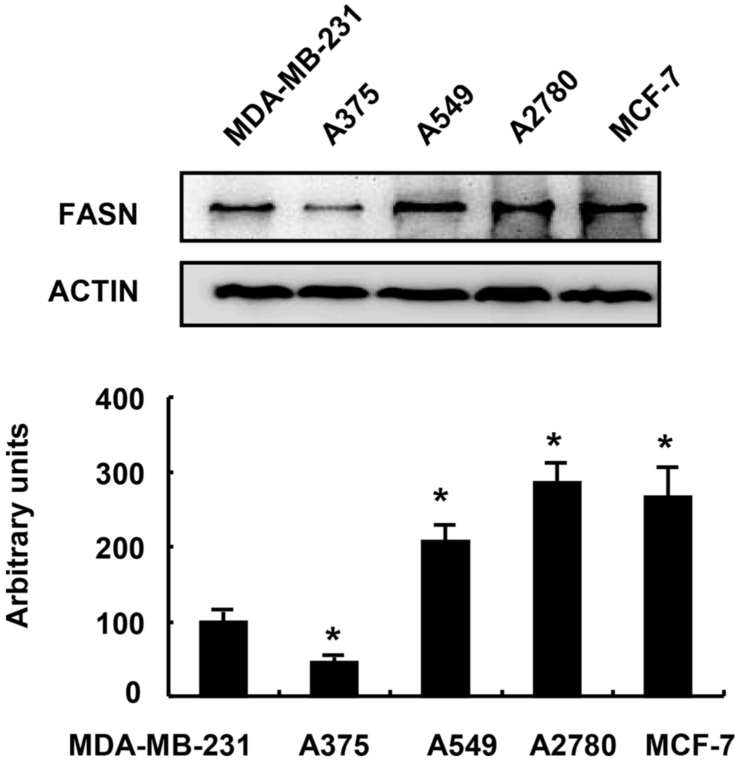

Supplementary Figure S5: FASN protein abundance in different cancer cells. FASN protein abundance was examined in the indicated cells. Means ± SEMs shown are representative of at least three independent *in vitro* experiments. Statistical significance was calculated using the two-tailed Student *t* test for the effects of the indicated cells vs. MDA-MB-231 cells (*$p < 0.05$).

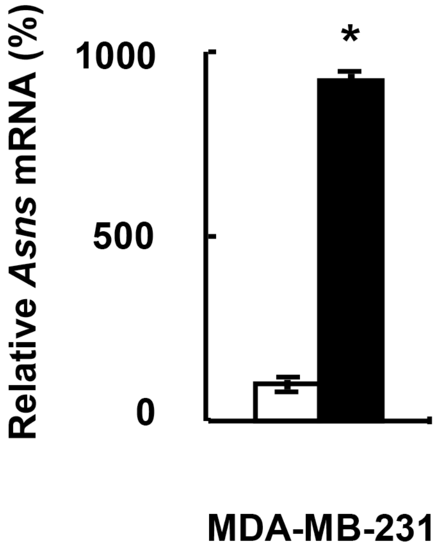

Supplementary Figure S6: The effect of leucine deprivation on *asparagine synthetase* (*Asns*) mRNA abundance. MDA-MB-231 cells were incubated in control (+leu) or leucine-deficient (−leu) medium for 48 h, followed by examination of the *Asns* mRNA abundance. Statistical significance was calculated using the two-tailed student *t* test for the effects of (−) leu vs. the control treatment (*$p < 0.05$).

List of oligonucleotide primer pairs used in RT-PCR analysis

Target gene	Forward Primer (5'-3')	Reverse Primer (5'-3')
Fasn	GCAAATTCGACCTTTCTCAGAAC	GGACCCCGTGGAATGTCA
Srebp1c	TGTGCAGACAGGGCCTTTG	CAGTGGGACTGTTGCCAAGA
Asns	TGAGAGGCTTCTGAGGGAACTC	ATGGGCAGCAGTAGTTCGATCT

Supplementary Figure S7: List of oligonucleotide primer pairs used in RT-PCR analysis.
